# Dietary probiotic ingestion is associated with elevated enterolignans concentration in the United States population, evidenced by NHANES 1999–2010

**DOI:** 10.3389/fnut.2025.1527029

**Published:** 2025-05-02

**Authors:** Jialong Dong, Liufang Huang, Chuchu Wang, Xinyi Luo, Jin Wei, KaiweiSa Abuduxukuer, Jianfeng Luo, Yifan Zhou, Qing Peng

**Affiliations:** ^1^Department of Ophthalmology, Shanghai Tenth People’s Hospital, School of Medicine, Tongji University, Shanghai, China; ^2^Division of Pediatric Gastroenterology and Nutrition, Xinhua Hospital, School of Medicine, Shanghai Jiao Tong University, Shanghai, China; ^3^NHC Key Laboratory of Health Technology Assessment, Fudan University, Shanghai, China; ^4^Key Laboratory of Public Health Safety, Ministry of Education, Fudan University, Shanghai, China; ^5^Department of Biostatistics, School of Public Health, Fudan University, Shanghai, China; ^6^School of Health and Rehabilitation Sciences, University of Pittsburgh, Pittsburgh, PA, United States; ^7^Department of Ophthalmology, Hospital of Chinese Traditional Medicine, Shanghai, China

**Keywords:** dietary, probiotics, enterolignan, microbiota, NHANES (National Health and Nutrition Examination Survey)

## Abstract

**Purpose:**

To investigate the associations between urinary enterolignans concentration and oral probiotic ingestion using nationally representative data from the United States population.

**Methods:**

We analyzed dietary recall data and urinary enterolignans concentrations from 12,358 eligible participants in the National Health and Nutrition Examination Survey (NHANES) 1999–2010. Linear regression models with comprehensive covariate adjustments were employed to assess associations, accounting for demographic, socioeconomic, health status, and lifestyle factors.

**Results:**

Participants with dietary probiotic ingestion had higher urinary concentrations of enterolignans, and probiotic ingestion showed robust and profound positive correlations with enterolignans after fully adjusted with multiple confounders (all *p* values<0.05). Frequent probiotic consumption exerts a more profound and positive impact on enterolignans concentrations than Infrequent probiotic consumption, according to correlation coefficient values in both univariate and multivariate analyses.

**Conclusion:**

Dietary probiotic consumption was significantly associated with elevated urinary enterolignans concentrations in the U.S. population, with high-frequency intake demonstrating a stronger dose–response relationship compared to low-frequency consumption.

## Introduction

Lignans, diphenolic compounds abundantly present in whole-grain cereals, seeds, and vegetables, represent the predominant phytoestrogens in traditional Western diets (1). Both ingested lignans and their microbial metabolites, enterolignans (including enterolactone [ENL] and enterodiol [END]), have been associated with diverse health benefits. Epidemiological evidence suggests these compounds may reduce risks of hormone-related cancers, cardiovascular mortality, type 2 diabetes, and all-cause mortality (2, 3). Their biological effects are primarily attributed to structural similarities with endogenous estrogens, enabling interactions with estrogen receptors (4).

**Figure 1:**
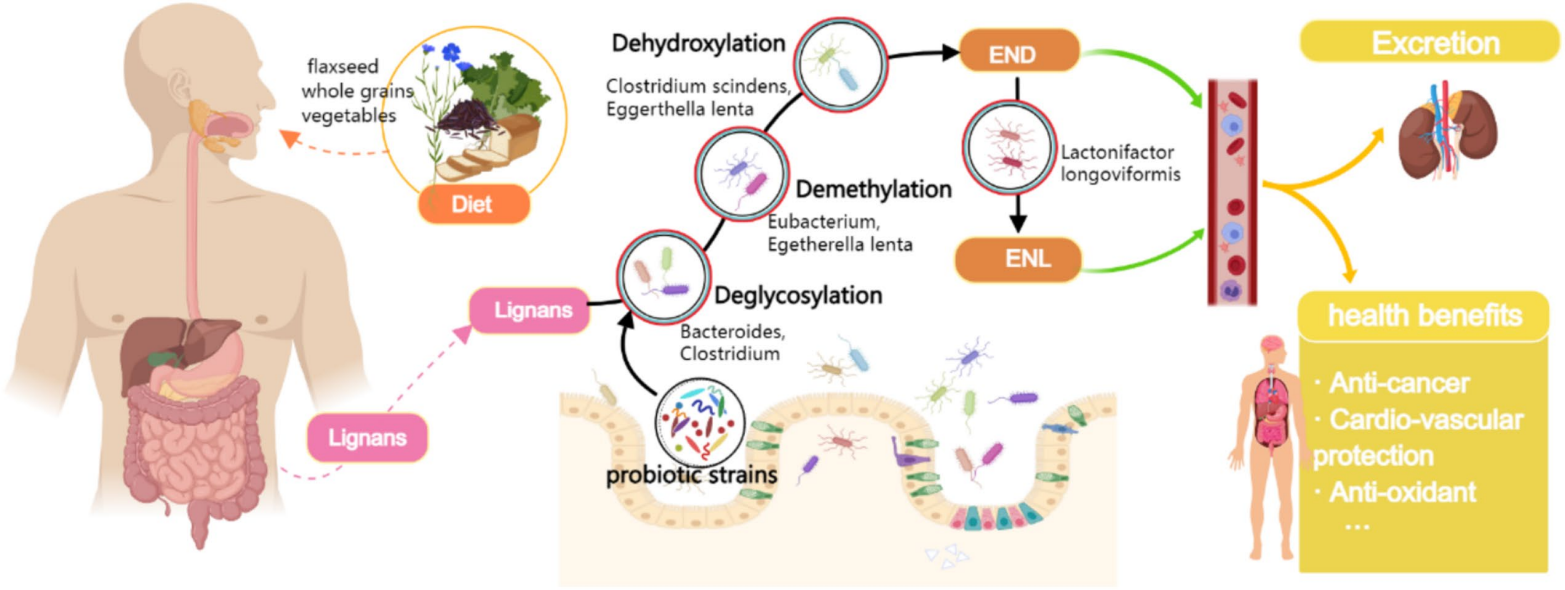
Biochemical pathway of dietary lignan metabolism to enterolignans (END/ENL) and the modulatory role of probiotic intake. Biochemical pathway of dietary lignan metabolism to enterolignans (enterodiol [END] and enterolactone [ENL]) mediated by gut microbiota. Key steps include deglycosylation, demethylation, dehydroxylation, and oxidation/reduction, catalyzed by specific bacterial taxa (labeled below each step). Probiotics (e.g., Lactobacillus and Bifidobacterium) may enhance metabolic flux by modulating microbiota composition or activity. Enterolignans are absorbed into systemic circulation, exerting health-promoting effects (Created with MedPeer.cn).

Notably, the bioavailability of dietary lignans in their native form is remarkably low. The conversion to bioactive enterolignans requires sequential metabolic transformations mediated by specific gut microbial communities (5) (Figure 1). Emerging evidence indicates that subdominant bacterial species within the gut microbiota play pivotal roles in this bioconversion process (6, 7). Importantly, circulating enterolignan levels - rather than dietary lignan intake per se - demonstrate more direct correlations with observed health benefits (8). This underscores the critical need to investigate factors modulating enterolignan production and interindividual variability in their systemic concentrations (9, 10).

Current research identifies multiple determinants of enterolignan levels, including age, gender, dietary patterns (particularly lignan-rich food consumption), antibiotic exposure, gut microbiota composition, body mass index (BMI), and smoking status (9). Notably, recent findings suggest a positive association between high enterolignan excretion and gut microbial diversity (11). Given the microbiota-dependent nature of enterolignan biosynthesis, dietary probiotic supplementation may represent a modifiable factor influencing their production and subsequent health effects. However, despite two decades of research on probiotics and gut microbiota modulation, the specific relationship between probiotic consumption and enterolignan concentrations remains underexplored.

To address this knowledge gap, we conducted a population-based study utilizing data from the National Health and Nutrition Examination Survey (NHANES) 1999–2010. This investigation represents the first large-scale epidemiological analysis examining the association between dietary probiotic intake and urinary enterolignan levels in a nationally representative U.S. cohort. Our findings provide novel insights into the potential of probiotic interventions to enhance enterolignan production, with implications for dietary strategies aimed at chronic disease prevention.

## Materials and methods

### Survey design and population

We obtained public datasets of the National Health and Nutrition Examination Survey (NHANES) from 1999 to 2010. Conducted by the National Center for Health Statistics (NCHS), the NHANES is a program of continuous and cross-sectional surveys on the health and nutritional status of noninstitutionalized U.S. adults. NHANES accepts a multistage, stratified sample design to represent the U.S. population according to demographic and geographic strata (12), and this sampling methodology ensures an accurate and diverse representation of the population. Therefore, secondary analytic findings from the NHANES datasets could be generalized to the broader U.S. population within certain statistical margins. NHANES datasets (1999–2010) were retrieved from the CDC’s public repository.^1^ Data extraction focused on variables including urinary enterolignans, dietary probiotic intake, and covariates (demographics, BMI, fiber intake). Keywords for variable selection included ‘probiotics,’ ‘yogurt,’ ‘enterolactone,’ and ‘enterodiol.’ The time range (1999–2010) was selected because urinary enterolignans were only measured during these survey cycles. The NCHS Research Ethics Review Board approved the study protocol of NHANES, and all respondents gave informed consent, which is publicly available online (See text footnote 1).

### Primary outcome: urinary enterolignans: enterodiol and enterolactone

**Figure 2:**
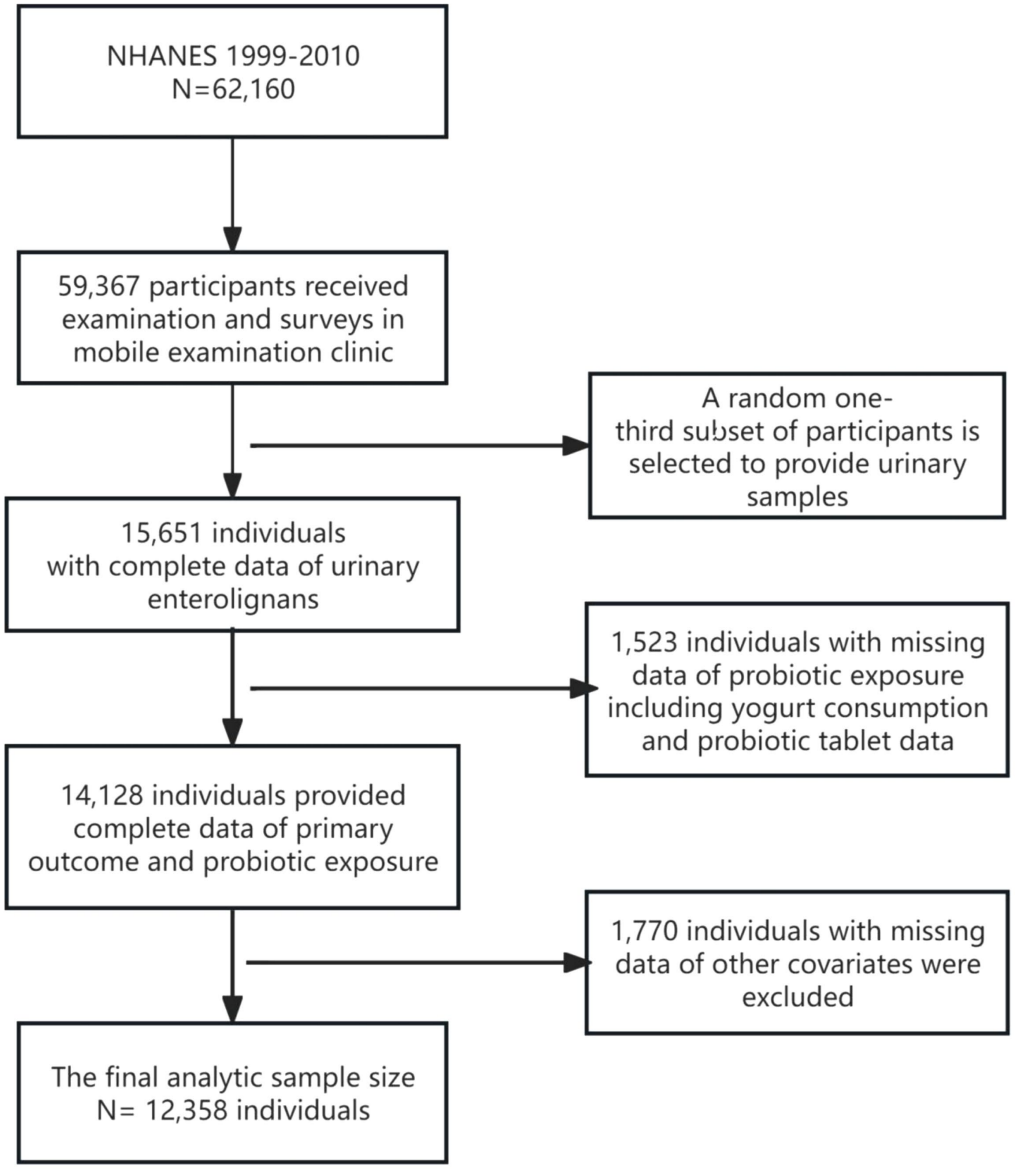
Flowchart of screening qualified participants for the observational study in NHANES 1999–2010.

The NHANES survey included a health examination at a mobile examination clinic/centre (MEC), which collected participants’ spot blood and urinary samples. The NHANES cycles from 1999 to 2010 were selected because urinary enterolignans (END and ENL) were exclusively measured during this period. Subsequent NHANES cycles discontinued these biomarker assessments, limiting data availability beyond 2010. This timeframe also ensures consistency in measurement protocols across survey years. According to NHANES 1999–2010, 62,160 participants were enrolled in the household interview, of which, 59,367 participants visited the MEC and a random one-third subset of participants was selected to provide biological specimens for biomarker measurement, including urinary ENL and END concentrations. Detailed protocols for collecting, processing, storing, shipping, and quantitative analysis of urinary specimens have been described previously (13–15). For the current study, 15,651 individuals with complete data on urinary enterolignans were included (Figure 2).

### Definition and classification of probiotic exposure

To sufficiently identify probiotic exposure, there has been a consensus that both dietary intake and daily supplements shall take into account (16–18), which requires both the Food Frequency Questionnaire (FFQ) and Dietary Supplement Use 30-Day Questionnaire (DSQ), to evaluate the yogurt and probiotic tablet consumption for a comprehensive picture of probiotic consumption. We analyzed the Dietary Supplement Use 30-day Study to assess the consumption of dietary supplements, including pre-and probiotics, during the 30 days before the NHANES interview. Detailed nonfood pre- or probiotic information has been described elsewhere (19) and could be verified through the public NHANES datasets.

Along with identifying yogurt consumers, we extracted the frequency of yogurt consumption from the FFQ. We classified yogurt consumers based on their weekly consumption into “High dose Yogurt,” defined as one time or more per week, and “Low dose Yogurt,” defined as yogurt consumption less than once a week. By this point, we classified ‘probiotic exposure’ into two groups: ‘High dose probiotic supplement’, which refers to ‘High dose Yogurt’ and/or ‘High dose probiotic tablet’, and ‘Low dose probiotic supplement’, which refers to ‘Low dose Yogurt’ and/or ‘Low dose probiotic tablet’.

### Study covariates

NHANES collects a wide range of demographic, socioeconomic, health condition and lifestyle-related data. Most factors contribute to the interpersonal variation of enterolactone concentrations reported by previous literature have been enrolled accordingly for the statistical analysis of the present study (9), which included age (years), gender (binary variable, male and female), race/ethnicity (non-Hispanic White, non-Hispanic Black, Hispanic, other race/multiracial), household poverty-to-income ratio (PIR, ≤1.85 as low; 1.85–3.5 as a medium; >3.5 as high), total energy (kcal/day) and fibre intake (g/day), and the adiposity indicator body mass index (BMI). After filtering participants with missing data from any covariate, 1,770 individuals were excluded from the analysis, which eventually led to a final statistical sample size of 12,358 (Figure 2).

### Statistical analyses

According to the NHANES dataset, the distributions of urinary enterolignans were highly right-skewed; therefore, we performed log transformation to adjust before parametric tests were performed. Such pre-processing of urinary enterolignans data has been widely accepted in related studies (13, 20). The dependent variable was the natural log transformation of END/ENL concentration. Chi-square tests were used to explore the distribution of categorical variables (sex, age, race, PIR) between groups with or without probiotic supplementation and between subgroups supplemented with probiotics. The *p*-value of χ^2^ tests was used to reflect the statistical significance between groups. We used the Satterthwaite-adjusted results in the Student *t*-test or the Wilcoxon rank-sum test to explore differences in the distribution of continuous variables (Total Energy Intake (kcal), Fiber Intake, BMI) between probiotic groups or subgroups. Simple linear regression was used to correlate each urine phytoestrogens, probiotic subgroups, or other covariates, reflecting the relationship between independent and dependent variables by estimated regression coefficients and 95% confidence intervals. In linear models, certain covariates have been log-transformed if needed to adjust non-normal distribution, and where both dependent and independent variables have been log-transformed, the dependent variable can be interpreted as percentage changes for a one-percent increase in the independent variable.

Multiple linear regressions were adjusted for demographic factors including age (<50 yrs., ≥50 yrs), gender and race (non-Hispanic White, non-Hispanic Black, Hispanic/Mexican American, other) as Model 1, plus a social-economic factor, PIR (≤1.85low, >1.85- ≤ 3.5 medium, >3.5 high) as Model 2; plus nutritional and dietary factors, including total energy and fiber intake, and the body mass index (BMI) which also represent metabolic and adiposity condition as Model 3. All models retained variables for uniform presentation and comparison of results across urinary phytoestrogens. Results were exhibited by the predicted regression coefficient and its 95% confidence interval in multiple linear regression, holding all other remaining covariates constant.

## Results

In total, 12,358 participants from NHANES 1999–2010 were deemed eligible for statistical analysis in the current study (Figure 2). A description of the characteristics distribution of the study sample according to dietary probiotic exposure is shown in Table 1. Compared to No probiotic supplement, participants with probiotic supplements had relatively higher END/ENL. There were no significant differences in END/ENL concentrations between the High-dose and Low-dose probiotic supplement groups (END, *p =* 0.0986; ENL, *p* = 0.1042).

**Table 1:** Characteristics of participants in the present study according to dietary probiotic exposure from NHANES 1999–2010.

Variables	Total	No supplement	Probiotic supplement	*p* value	Low dose probiotic supplement	High dose probiotic supplement	*p* value
Gender				<0.0001			0.0106
Male	5,974 (48.34)	5,018 (49.89)	956 (41.58)		637 (43.57)	319 (38.11)	
Female	6,384 (51.66)	5,041 (50.11)	1,343 (58.42)		825 (56.43)	518 (61.89)	
Age				<0.0001			0.8959
<50	8,668 (70.14)	6,934 (68.93)	1734 (75.42)		1,104 (75.51)	630 (75.27)	
≥50	3,690 (29.86)	3,125 (31.07)	565 (24.58)		358 (24.49)	207 (24.73)	
Race				0.8569			0.0144
Mexican American	2,956 (23.92)	2,372 (23.58)	584 (25.40)		360 (24.62)	224 (26.76)	
Other Hispanic	759 (6.14)	677 (6.73)	82 (3.57)		49 (3.35)	33 (3.94)	
Non-Hispanic White	5,284 (42.76)	4,258 (42.33)	1,026 (44.63)		627 (42.89)	399 (47.67)	
Non-Hispanic Black	2,847 (23.04)	2,353 (23.39)	494 (21.49)		353 (24.15)	141 (16.85)	
Other Race - Including Multi-Racial	512 (4.14)	399 (3.97)	113 (4.92)		73 (4.99)	40 (4.78)	
PIR				<0.0001			0.0374
Low	5,759 (46.60)	4,787 (47.59)	972 (42.28)		625 (42.75)	347 (41.46)	
Medium	3,053 (24.70)	2,480 (24.65)	573 (24.92)		392 (26.81)	181 (21.62)	
High	3,546 (28.69)	2,792 (27.76)	754 (32.80)		445 (30.44)	309 (36.92)	
Total Energy Intake (kcal)	2081.17 ± 901.61	2083.66 ± 925.62	2070.25 ± 788.10	<0.0001	2100.922 ± 831.53	2016.67 ± 703.13	0.1155
Fiber intake	15.04 ± 8.61	15.00 ± 8.79	15.26 ± 7.80	<0.0001	14.67 ± 7.29	16.27 ± 8.52	<0.0001
BMI	26.29 ± 7.19	26.41 ± 7.20	25.78 ± 7.15	0.0002	26.18 ± 7.17	25.07 ± 7.06	0.0003
Enterodiol^1^	3.64 ± 2.52	3.46 ± 1.59	3.70 ± 1.55	<0.0001	3.66 ± 1.56	3.77 ± 1.54	0.0986
Enterolactone^1^	5.84 ± 2.84	5.51 ± 1.70	5.78 ± 1.59	<0.0001	5.74 ± 1.61	5.85 ± 1.56	0.1042

^1Logarithmic transformation.^

**Figure 3:**
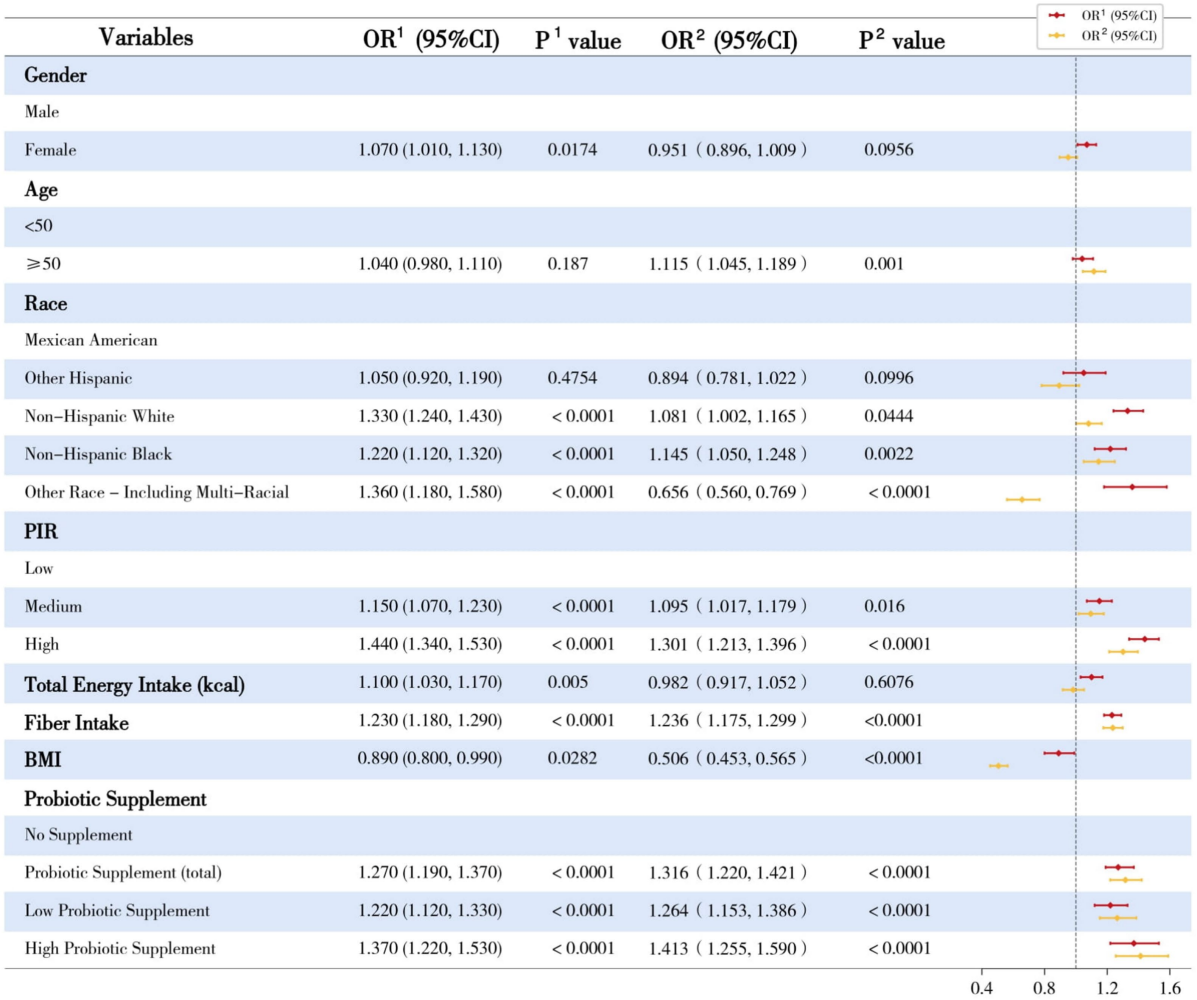
Univariate linear regression analyses of dependent variates and enterolignans. OR, odds ratio; 95%CI, 95% confidence interval; ^1^Enterodiol; ^2^Enterolactone; PIR, Poverty Income Ratio; BMI, Body Mass Index. This forest plot illustrates the univariate linear regression analyses of urinary enterolignans (enterodiol [END] and enterolactone [ENL]) concentrations across various demographic, socioeconomic, and dietary factors. The plot presents the regression coefficients (*β*) and their corresponding 95% confidence intervals (CIs) for each variable.

Bivariate methods were used to describe individual demographic, socioeconomic, dietary, and lifestyle variables’ associations with urinary enterolignan concentrations (Figure 3). Age, gender, and race-ethnicity had significant and differential associations with END or ENL. Participants with higher incomes appeared to have higher concentrations of both END and ENL (all *p* values < 0.05). Dietary factors, especially dietary fiber intake, had a profound and positive association with urinary enterolignans. The adiposity indicator, BMI, on the other hand, had a negative association with both urinary enterolignans (END: OR = 0.89, 95% CI: [0.80, 0.99], *p* = 0.0282; ENL: OR = 0.5058, 95% CI: [0.4527, 0.5649], *p* < 0.0001). Probiotic ingestion had significant and positive associations with both END and ENL (all *p* values < 0.0001). Higher-dose probiotic supplements exhibited a more substantial positive impact on enterolignans than low-dose probiotic supplements, according to comparisons of OR values (Figure 3).

Multiple regression models were used to verify further the independent correlations between dietary probiotic ingestion and urinary enterolignans. After adjusting for various covariates, the associations between dietary probiotic supplements and enterolignans maintained their significance (all *p* values <0.05, Table 2). Consistent with the univariate results, High dose probiotic supplements had more significant beta coefficients than Low dose probiotic supplements (fully adjusted model: END, *β* values: 0.1793 versus 0.2432; END, *β* values: 0.2334 versus 0.2700, Table 2).

**Table 2:** Multivariate linear regression analyses of dietary probiotic ingestion and enterolignans.

Probiotic ingestion	Enterodiol (ln-ng/mg)					
	Model 1		Model 2		Model 3	
	β	95%CI	β	95%CI	β	95%CI
Total probiotic ingestion	0.2347**	(0.1628, 0.3067)	0.2197**	(0.1479, 0.2916)	0.2023**	(0.1307, 0.2740)
No supplement	Ref					
Low dose probiotic supplement	0.1951**	(0.1082, 0.2820)	0.1843**	(0.0976, 0.2710)	0.1793**	(0.0928, 0.2657)
High dose probiotic supplement	0.3043**	(0.1925, 0.4161)	0.2820**	(0.1704, 0.3936)	0.2432**	(0.1316, 0.3547)

	Enterolactone (ln-ng/mg)					
	Model 1		Model 2		Model 3	
	β	95%CI	β	95%CI	β	95%CI
Total probiotic ingestion	0.2893**	(0.2129, 0.3657)	0.2765**	(0.2002, 0.3528)	0.2466**	(0.1711, 0.3221)
No supplement	Ref					
Low dose probiotic supplement	0.2452**	(0.1530, 0.3375)	0.2364**	(0.1442, 0.3285)	0.2334**	(0.1424, 0.3244)
High dose probiotic supplement	0.3666**	(0.2480, 0.4853)	0.3471**	(0.2286, 0.4657)	0.2700**	(0.1525, 0.3875)

The results of the multivariable linear regression models were expressed as β and 95% confidence intervals (CI). The analytic sample size was 12,358.

Model 1: adjusted for demorgraphic factors including age, gender and race; Model 2: adjusted for factors in Model 1, as well as social-economic factor, PIR; Model 3: adjusted for factors in Model 2, as well as nutritional and dietary factors, including total energy intake and dietary fiber intake, and body mass index (BMI) which also represent metabolic and adiposity condition.

*p < 0.05; **p < 0.005.

Subgroup analyses were further performed on antimicrobial users to investigate the role of homeostasis and disturbance of the intestinal environment and microbiota. To our surprise, in antimicrobial users, dietary probiotic ingestion remained significantly correlated to END and ENL, even after adjustments of various covariates (all *p* values <0.05, Table 3).

**Table 3:** Robust test for multivariate linear regression analyses in antimicrobial users.

Probiotic ingestion	Enterodiol (ln-ng/mg)					
	Model 1		Model 2		Model 3	
	β	95%CI	β	95%CI	β	95%CI
Total probiotic ingestion	0.1946**	(0.1040, 0.2852)	0.1871**	(0.0966, 0.2775)	0.1671*	(0.0767, 0.2575)
No supplement	Ref					
Low dose probiotic supplement	0.1563*	(0.0464, 0.2662)	0.1503*	(0.0405, 0.2600)	0.1405*	(0.0309, 0.2500)
High dose probiotic supplement	0.2597*	(0.1204, 0.3991)	0.2497*	(0.1105, 0.3889)	0.2127*	(0.0734, 0.3521)

	Enterolactone (ln-ng/mg)					
	Model 1		Model 2		Model 3	
	β	95%CI	β	95%CI	β	95%CI
Total probiotic ingestion	0.2737**	(0.1783, 0.3691)	0.2666**	(0.1713, 0.3619)	0.2272**	(0.1328, 0.3216)
No supplement	Ref					
Low dose probiotic supplement	0.2490**	(0.1332, 0.3648)	0.2443**	(0.1286, 0.3600)	0.2306**	(0.1162, 0.3449)
High dose probiotic supplement	0.3156**	(0.1688, 0.4625)	0.3046**	(0.1578, 0.4513)	0.2215*	(0.0760, 0.3670)

The analytic sample size was 7,321.

Model 1: adjusted for demorgraphic factors including age, gender and race; Model 2: adjusted for factors in Model 1, as well as social-economic factor, PIR; Model 3: adjusted for factors in Model 2, as well as nutritional and dietary factors, including total energy intake and dietary fiber intake, and body mass index (BMI) which also represent metabolic and adiposity condition.*p < 0.05; **p < 0.005.

## Discussion

The current literature is insufficient to reach definitive recommendations for dietary live microbe intake for beneficial health outcomes, lacking objective and bio-molecular evidence. Phytoestrogens such as lignans and their *in vivo* metabolites, including enterolignans (e.g., enterodiol [END] and enterolactone [ENL]), have been widely recognized for their health benefits. Therefore, the current study provides the first Epidemiology evidence of a positive correlation between probiotic supplements and health-beneficial metabolites derived from dietary intake and gut microbiota.

Lignans are the predominant class of phytoestrogens in the Western diet (5), therefore, the health-beneficial effects of enterolignans are largely dependent on their in *vivo* concentrations, which are primarily determined by the conversion of dietary lignans to enterolignans. The composition and activity of the gut microbiota appear to be the most critical factors governing inter-individual variability in circulating enterolignan concentrations (9).

*In vitro* studies have demonstrated that the conversion of lignans to enterolignans requires abundant and viable bacteria (21). The metabolism of dietary lignans, including secoisolariciresinol diglycoside, lariciresinol, matairesinol, and pinoresinol, to END involves multiple biochemical processes, such as deglycosylation, demethylation, and dehydroxylation (5). Several strains of *Bacteroides* and *Clostridium* have been identified to catalyze the deglycosylation step (16), while demethylation is catalyzed by *Butyribacterium methylotrophicum*, *Eggerthella lenta*, *Peptostreptococcus productus*, *Eubacterium*, and *Enterococcus faecalis* (17, 18). The dehydroxylation process is catalyzed by *Clostridium scindens* strains and *Eggerthella lenta* (22). END can also be converted to ENL via dehydrogenation catalyzed by specific bacteria such as *Lactonifactor longoviformis* (16). A summary of the enhanced abundance of specific intestinal enterolignans-converting bacteria mentioned above by ingestive probiotics or yogurt consumption in humans has been listed in Supplementary Table 1.

A recent epidemiological study reported that lower serum ENL concentrations correlate with lower fecal bacterial counts, especially those from the *Lactobacillus* and *Enterococcus* groups (23). Based on the present study, higher-dose probiotic consumption appears to have a more profound impact on END/ENL concentrations compared to lower-dose consumption, as indicated by the beta coefficient values in both univariate and multivariate analyses (Figure 3; Table 2). We therefore hypothesize that the elevated END/ENL concentrations may be attributed to the increased abundance of enterolignan-converting bacteria in the gut due to dietary probiotic intake. Probiotic supplements may directly enhance the abundance of beneficial strains in the gut or indirectly promote the proliferation of specific bacteria. So this study support the integration of probiotic-rich foods such as yogurt or evidence-based probiotic supplements into daily dietary patterns as a practical strategy to contribute to health benefits. Future research should focus on establishing causal relationships between probiotic intake and enterolignan levels, while also exploring the specific mechanisms and long-term health impacts of this association.

Lignan-rich diets (e.g., whole grains, seeds) provide dietary source for enterolignan synthesis, therefore, dietary factors warrant careful consideration in this analysis. Lignans predominantly exist in fiber-rich plant sources such as whole grains, seeds, and vegetables (21). This intrinsic association enables epidemiological studies to estimate lignan exposure through fiber intake data when direct quantification is unavailable. Also, Dietary fibers are crucial for gut microbiota as they serve as nutrient sources for gut microorganisms, modulating microbial diversity and function (24). As such, nutritional factors like dietary pattern, especially the fiber/lignan intake, may confound the conclusion of probiotic’s impacts on enterolignan elevation. To address this, our analysis further enrolled Total Energy Intake and dietary fiber intake (representative as proxies for lignan-containing foods and dietary pattern) for adjustment in statistical models. Notably, the robust association between probiotic use and elevated enterolignans persisted even after these adjustments (Table 2, Model 3). So we can say that, the probiotic ingestion in modulating this process might be independent of dietary pattern, or specifically, the direct lignan/fiber in the dietary intake. However, ethnic variations in dietary patterns and host genetics may introduce heterogeneity in enterolignan metabolism. Future studies should incorporate direct lignan quantification and metagenomic profiling to disentangle diet-microbiome-host interactions in enterolignan biosynthesis.

Antibiotic use has been reported to correlate to lower END (13, 20, 25). The duration of decreased ENL owing to oral antimicrobial use may last for up to 12–16 months (25), and the reason for lower enterolignans with antimicrobial use is apparently and primarily attributed to the disturbed intestinal microbiota (9, 25), which limits the production of enterolignans. Therefore, we performed a subgroup analysis in which participants reported positive for antimicrobial tablet use in the previous 30 days. It is noteworthy that the positive associations between ingestive probiotics and enterolignans remained robust across multple adjusting models (Table 3). As such, we assume that probiotic ingestion elevates enterolignans concentration via its positive impacts on the abundance of specific intestinal microbiota for enterolignans production even after the disturbance of the intestinal environment by antimicrobial use. These findings hold significant clinical implications for mitigating antibiotic-induced microbiota dysbiosis and accelerating the restoration of enterolignan-producing microbial consortia during post-antibiotic recovery. However, the dual roles of probiotic interventions necessitate cautious interpretation. For example, a recent study have demonstrated that excessive proliferation of *Akkermansia muciniphila*—a next-generation probiotic—can enhance the virulence and infection potential of enteric pathogens (26). Given the potential adverse effects of probiotics, such as diarrhea and infections, particularly in vulnerable populations (27, 28), their use should be judiciously tailored to individual needs. Furthermore, regulatory standardization of probiotic formulations is essential to ensure product consistency and clinically translatable outcomes.

### Strengths and limitations

Our study has several strengths which need to be highlighted. First, we analyzed a large, multi-ethnic, and national survey representative of the US population to generate real-world evidence. To the best of our knowledge, this is the first study to provide empirical and quantified evidence of the positive impacts of dietary probiotic ingestion on specific health-beneficial dietary- and gut microbiota-derived metabolites. Given the high proportion of dietary probiotic users in the US population, according to the current study and previous literature, our results may support a simple and practical approach to enhance the health effects of enterolignans. Multiple confounders were further adjusted in the multi-variates linear regression to assess the robustness of such positive associations.

On the other hand, several limitations must be acknowledged as well. Firstly, the cross-sectional nature of our research may merely indicate associations, which was methodologically challenging. Secondly, the methods used to estimate microbial content intake was evaluated within the last 30 days food recall questionnaire, which may not fully represent the long-term nutritional supplement, and the questionnaire data may also encounter recall bias. Moreover, the NHANES cohort’s U.S.-centric design limits generalizability to other ethnicities and regions. Global variations in diet, gut microbiome, and genetic polymorphisms affecting lignan metabolism may alter observed associations, necessitating future multi-ethnic studies with standardized enterolignan measurements for validation. Finally, future studies should employ longitudinal designs to establish causality, identify specific probiotic strains enhancing enterolignan production, investigate gut microbiota-mediated mechanisms, and validate findings across diverse populations through randomized controlled trials.

## Conclusion

This study demonstrates a significant positive association between dietary probiotic intake and elevated urinary enterolignan concentrations in a nationally representative U.S. population. Notably, high-frequency probiotic consumption exhibited stronger associations than low-frequency intake, and robustness persisted even among antimicrobial users. The findings provide scientific evidence for incorporating probiotic-enriched dietary interventions or supplementary regimens to enhance enterolignan biosynthesis and optimize its physiological concentrations. Future longitudinal and mechanistic studies are warranted to establish causality, identify strain-specific effects, and refine microbiota-targeted dietary recommendations.

## Data Availability

The original contributions presented in the study are included in the article/Supplementary material, further inquiries can be directed to the corresponding authors.

## Footnotes

^1^ https://www.cdc.gov/nchs/nhanes/
